# Development of parallel measures to assess HIV stigma and discrimination among people living with HIV, community members and health workers in the HPTN 071 (PopART) trial in Zambia and South Africa

**DOI:** 10.1002/jia2.25421

**Published:** 2019-12-16

**Authors:** Anne L Stangl, Pamela Lilleston, Hlengani Mathema, Triantafyllos Pliakas, Shari Krishnaratne, Kirsty Sievwright, Nomhle Bell‐Mandla, Redwaan Vermaak, Tila Mainga, Mara Steinhaus, Deborah Donnell, Ab Schaap, Peter Bock, Helen Ayles, Richard Hayes, Graeme Hoddinott, Virginia Bond, James R Hargreaves, on behalf of the HPTN 071 (PopART) Study Team

**Affiliations:** ^1^ International Center for Research on Women Washington DC USA; ^2^ Desmond Tutu TB Centre Department of Paediatrics and Child Health Faculty of Medicine and Health Sciences Stellenbosch University Cape Town South Africa; ^3^ Division of Public Health Surveillance and Response National Institute for Communicable Diseases National Health Laboratory Service Johannesburg South Africa; ^4^ London School of Hygiene and Tropical Medicine London United Kingdom; ^5^ Zambart School of Medicine University of Zambia Lusaka Zambia; ^6^ SCHARP Seattle Washington USA

**Keywords:** HIV, stigma, discrimination, measurement, Sub‐Saharan Africa, antiretroviral therapy

## Abstract

**Introduction:**

Integrating standardized measures of HIV stigma and discrimination into research studies of emerging HIV prevention approaches could enhance uptake and retention of these approaches, and care and treatment for people living with HIV (PLHIV), by informing stigma mitigation strategies. We sought to develop a succinct set of measures to capture key domains of stigma for use in research on HIV prevention technologies.

**Methods:**

From 2013 to 2015, we collected baseline data on HIV stigma from three populations (PLHIV (N = 4053), community members (N = 5782) and health workers (N = 1560)) in 21 study communities in South Africa and Zambia participating in the HPTN 071 (PopART) cluster‐randomized trial. Forty questions were adapted from a harmonized set of measures developed in a consultative, global process. Informed by theory and factor analysis, we developed seven scales, with values ranging from 0 to 3, based on a 4‐point agreement Likert, and calculated means to assess different aspects of stigma. Higher means reflected more stigma. We developed two measures capturing percentages of PLHIV who reported experiencing any stigma in communities or healthcare settings in the past 12 months. We validated our measures by examining reliability using Cronbach's alpha and comparing the distribution of responses across characteristics previously associated with HIV stigma.

**Results:**

Thirty‐five questions ultimately contributed to seven scales and two experience measures. All scales demonstrated acceptable to very good internal consistency. Among PLHIV, a scale captured internalized stigma, and experience measures demonstrated that 22.0% of PLHIV experienced stigma in the community and 7.1% in healthcare settings. Three scales for community members assessed fear and judgement, perceived stigma in the community and perceived stigma in healthcare settings. Similarly, health worker scales assessed fear and judgement, perceived stigma in the community and perceived co‐worker stigma in healthcare settings. A higher proportion of community members and health workers reported perceived stigma than the proportion of PLHIV who reported experiences of stigma.

**Conclusions:**

We developed novel, valid measures that allowed for triangulation of HIV stigma across three populations in a large‐scale study. Such comparisons will illuminate how stigma influences and is influenced by programmatic changes to HIV service delivery over time.

## 1 Introduction

A number of efficacious technologies are now available to prevent HIV transmission 1, 2, 3, however, their population impact will depend upon the public's capacity to take up and adhere to these technologies 4, 5. HIV stigma and discrimination are common barriers to HIV prevention, care and treatment services, including HIV testing, condom use, adherence to antiretroviral therapy (ART), and uptake of prevention‐of‐mother‐to‐child transmission services 6, 7. Fear of experiencing stigma has also been shown to hinder product use among young women participating in microbicide and pre‐exposure prophylaxis trials in sub‐Saharan Africa, potentially contributing to the failure of these technologies to protect participants from acquiring HIV 8, 9, 10.

Measuring HIV stigma and discrimination in the context of an HIV prevention intervention requires assessment of stigma among at least three populations: people living with HIV (PLHIV), community members (CMs) in the areas where the biomedical intervention is being implemented, and health workers (HWs) delivering the intervention. Researchers have suggested that using “parallel measures” to assess stigma across these populations will enable the most complete assessment of stigma by allowing for triangulation of data across population groups 11, 12. For example, a South African study developed scales comparing “blame and judgement” and “interpersonal distancing” among CMs with “self‐stigma” and “expected stigma” among PLHIV 13. Despite the value of this approach, no studies have utilized parallel measures to examine the influence of HIV stigma on biomedical outcomes, such as HIV incidence and treatment adherence, in a trial setting. Having validated, parallel measures to assess stigma across PLHIV, CMs and HWs is a critical first step to facilitating such research.

More common are scales assessing different domains of stigma separately among specific populations, including CMs 14, 15, 16, 17, 18, 19, 20, PLHIV 21, 22, 23, 24, 25, 26, 27 and HWs 28, 29, 30, 31, 32, 33, 34, 35, 36. While such measures may provide an in‐depth assessment of stigma in a particular population or setting, they may be less able to elucidate how stigma operates in the context of a population‐level biomedical trial with both community and health setting components. Key domains of stigma that can be shifted through a trial linked intervention are not always clearly defined. Additionally, the domains are not measured consistently across trials and other studies, limiting comparisons and meta‐analyses. Consequently, measures of stigma are not developed to encourage triangulation of perceptions, attitudes and experiences across different populations 37.

In this study, we sought to address these limitations by developing parallel survey questions from a brief set of items that emerged following a five‐year global process to articulate a practical theoretical framework of the stigmatization process in the context of HIV and derive standardized indicators of HIV stigma and discrimination 11, 38. We then explored the measures in the context of the HIV Prevention Trials Network (HPTN) 071 (PopART) trial, which tested the impact of a combination HIV prevention package, including universal door‐to‐door HIV testing and an offer of ART regardless of CD4 count, on HIV incidence in 21 urban communities in Zambia and South Africa (seven matched triplets, three in South Africa and four in Zambia) 39. Our goals were to develop a succinct set of valid and reliable measures to capture key domains of stigma for use in research on HIV prevention technologies, and to describe and compare the distribution of the resulting stigma measures across PLHIV, CMs and HWs, leveraging our unique opportunity to capture data across these three populations.

## 2 Methods

### 2.1 Stigma domains assessed

The key domains of the stigmatization process measured were derived from “Reducing HIV stigma and discrimination: a framework for programme implementation and measurement” 40, which has more recently been broadened to encompass health‐related stigma and discrimination, including HIV 41. We set out to measure the *drivers* (i.e. attitudes, such as shame of association with and judgement towards PLHIV among CMs and HWs) and *manifestations* (i.e. *internalized* stigma – the application of stigma to oneself 42, 43 – and *experienced* stigma 7, 44 among PLHIV and *perceived* stigma – a person's understanding of how others may act towards, think, or feel about someone living with HIV 26, 43] – among CMs and HWs) of HIV stigma 41.

### 2.2 Study populations, design and data collection

We collected data in Zambia and South Africa among participants in the HPTN 071 (PopART) trial and HWs implementing the intervention to simultaneously assess HIV stigma among three distinct populations: PLHIV, CMs and HWs (Figure S1). Baseline survey data were collected from PLHIV and CMs enrolled in the population cohort (PC) of the HPTN 071 (PopART) trial between November 2013 and March 2015. The PC consisted of approximately 2000 men and women between the ages of 18 and 44 randomly selected from each of the 21 communities to complete an interviewer‐administered questionnaire 39 in their local language. Participants were asked to self‐report their HIV status, and those who reported a positive HIV status completed an additional module on HIV stigma. HIV status was later confirmed by testing blood samples drawn from consenting survey participants as previously described 45. In this analysis, we included data on self‐reported internalized and experienced stigma among participants in the PC who self‐reported living with HIV and had a confirmed HIV positive test result. We also included data from a random sample of 20% of PC participants from each community who received a series of questions on HIV stigma‐related attitudes and perceptions. Participants living with HIV were excluded from this community member sample.

We also collected baseline data from HWs in study communities between July 2014 and June 2015. HWs included health facility staff (e.g. doctors, nurses, pharmacists, counsellors, security guards) working in study communities, Community HIV‐care Providers (CHiPs) employed by PopART to deliver the combination prevention package in the community, and other community‐based health workers (CHWs), not employed by PopART, involved in HIV services in both countries 46. HWs who self‐reported that they were living with HIV were not included in this analysis. Due to the low frequency of missing values across the three datasets (2 to 13%), our large sample size, and the nature of the questions, for which we deemed imputation inappropriate, individuals missing one or more items in each scale were dropped from analyses. Analyses confirmed that respondents with missing data on the stigma questions were not significantly different from respondents with complete data on the stigma questions based on socio‐demographic characteristics (data not shown).

### 2.3 Scale and experience measure development

**Figure 1:**
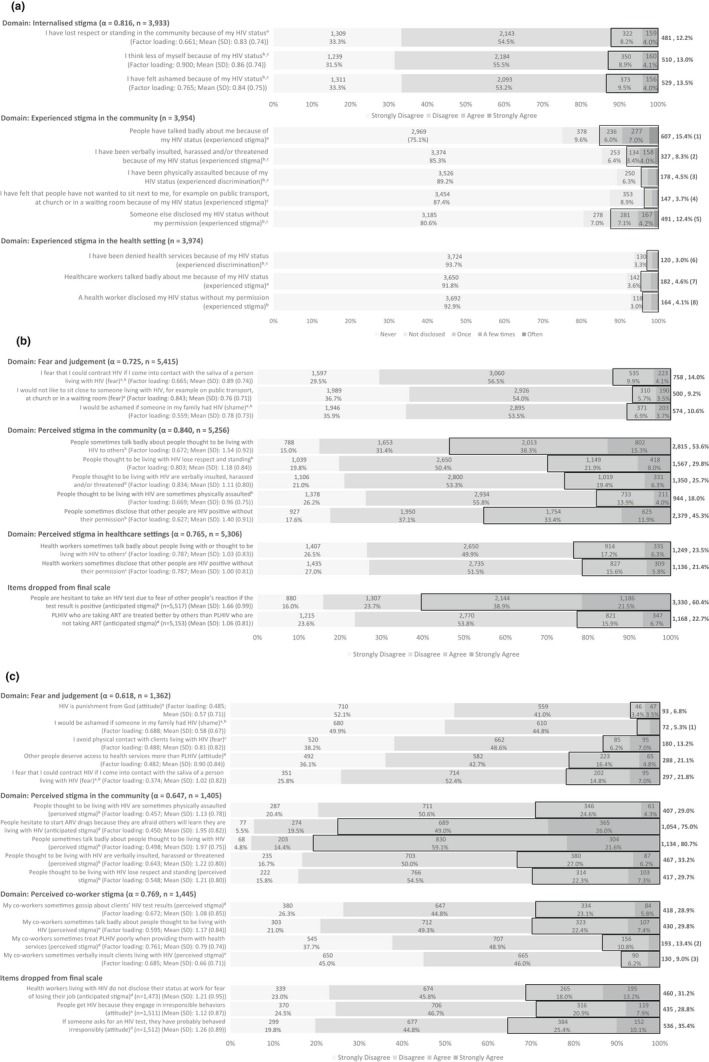
a, Responses to, and sources of HIV stigma items asked of people living with HIV. Agree or strongly agree for internalised stigma and experienced stigma at least once in the community or healthcare settings highlighted in bold. (1) Often: 94 (2.4%); (2) Often: 35 (0.9%); (3) Once: 70 (1.8%), A few times: 86 (2.2%), Often: 22 (0.6%); (4) Once: 81 (2.0%), A few times: 49 (1.2%), Often: 17 (0.4%); (5) Often: 43 (1.1%); (6) Once: 56 (1.4%), A few times: 51 (1.3%), Often: 13 (0.3%); (7) Once: 102 (2.6%), A few times: 67 (1.7%), Often: 13 (0.3%); (8) Once: 91 (2.3%), A few times: 64 (1.6%), Often: 9 (0.2%). ^a^Adapted from new item included in the 2015 to 2020 DHS Standard Survey instrument (https://dhsprogram.com/pubs/pdf/DHSQ7/DHS7-Womans-QRE-EN-07Jun2017-DHSQ7.pdf); ^b^PLHIV Stigma Index survey (http://www.stigmaindex.org/about-index); ^c^USAID stigma measures (https://www.icrw.org/wp-content/uploads/2016/10/Working-Report-Measuring-HIV-Stigma-Results-of-a-Field-Test-in-Tanzania.pdf). b, Responses to, and sources of, HIV stigma items asked of community members. Agree or strongly agree highlighted in bold. ^a^USAID stigma measures (https://www.icrw.org/wp-content/uploads/2016/10/Working-Report-Measuring-HIV-Stigma-Results-of-a-Field-Test-in-Tanzania.pdf); ^b^2015 to 2020 DHS Standard Survey Instrument (https://dhsprogram.com/pubs/pdf/DHSQ7/DHS7-Womans-QRE-EN-07Jun2017-DHSQ7.pdf); ^c^Adapted from new item included in the 2015 to 2020 DHS Standard Survey instrument; ^d^New item. c, Responses to, and sources of, HIV stigma items asked of health workers. Agree or strongly agree highlighted in bold. (1) Agree: 41 (3.0%), Strongly Agree: 31 (2.3%); (2) Strongly Agree: 37 (2.6%); (3) Strongly Agree: 40 (2.8%). ^a^USAID stigma measures (https://www.icrw.org/wp-content/uploads/2016/10/Working-Report-Measuring-HIV-Stigma-Results-of-a-Field-Test-in-Tanzania.pdf); ^b^2015 to 2020 DHS Standard Survey Instrument (https://dhsprogram.com/pubs/pdf/DHSQ7/DHS7-Womans-QRE-EN-07Jun2017-DHSQ7.pdf); ^c^Nyblade et al. A brief, standardized tool for measuring HIV‐related stigma among health facility staff: results of field testing in China, Dominica, Egypt, Kenya, Puerto Rico and St. Christopher and Nevis. 2013, J Int AIDS Soc; ^d^New item; ^e^Adapted from new item included in the 2015 to 2020 DHS Standard Survey instrument (https://dhsprogram.com/pubs/pdf/DHSQ7/DHS7-Womans-QRE-EN-07Jun2017-DHSQ7.pdf)

Item selection and development of scales and experience measures were informed by analysis in four phases. In the first phase, desk review and expert consultation were used to identify HIV stigma items for inclusion in the PLHIV, community member, and health worker questionnaires and establish preliminary face validity 47. Some items were derived from previously published and validated measures 33, 48, 49, while others were developed based on consultation with experts from the HIV stigma field (Figure 1a‐c). We used 11 items assessing HIV stigma among PLHIV, three of which captured internalized stigma and used a 4‐point agreement rating scale from 0= “strongly disagree” to 3= “strongly agree” (Figure 1a). The other eight items captured the frequency of occurrence (never, not disclosed, once, a few times, often) of reported experienced stigma in the community (five items) or the healthcare setting (three items) (Figure 1a). For the HIV fear and judgement scale among CMs and HWs, items reflected commonly reported drivers like fear of HIV infection (I fear that I could contract HIV if I come into contact with the saliva of a person living with HIV), shame (I would be ashamed if someone in my family had HIV) and judgement (HIV is punishment from God).

The most common or severe forms of stigma and discrimination reported by PLHIV in the literature and through expert consultation informed the items selected to assess stigma perceived by CMs and HWs. For example, CMs and HWs were asked whether “people thought to be living with HIV were verbally insulted, harassed, and/or threatened” and whether “people thought to be living with HIV lose respect and standing.” Where possible, items were adapted for parallel use across the three populations. For example, while CMs responded to the item “people sometimes talk badly about people living with HIV,” HWs responded to the item “my co‐workers sometimes talk badly about people living with HIV” and PLHIV responded to the item “people have talked badly about me because of my HIV status.”

The questionnaires originally included 12 items for CMs not living with HIV (Figure 1b) and 17 items for HWs (Figure 1c), which we considered including in the measures. In developing the scales, we chose to drop two of the items asked of CMs and three of the items asked of HWs, informed by the literature and expert consultation. Three of the five items captured distinct aspects of anticipated stigma related to HIV testing, ART use, (Figure 1b) and disclosure of HIV status at work (Figure 1c) that we decided should not be combined with items assessing stigma related to living with HIV. The two additional health worker items dropped assessed perceptions of PLHIV as being “irresponsible.” As these items were not asked of CMs, we decided to drop them in order to retain the parallel nature of the measures across the two populations.

In the second phase, we conducted factor analysis to assess the underlying domains of stigma and reduce the number of items needed to assess them 50. While we anticipated the domains that would be represented based on our previous research and measurement work 40, we used exploratory factor analysis as some of the survey items were new or not previously validated. Initially, all items asked of a specific study population were included. Eigenvalues were generated and scree plots visually inspected to suggest the number of factors that explained most of the variability in the data for each set of items 51. To examine the loading strength of items on each factor, we used iterated principal factor estimation 50 with oblique (promax) rotation, which allows for correlation between factors, as we expected the underlying domains of stigma to be correlated. The item groupings that emerged were generally consistent with the expected domains of stigma for all three populations. In the few instances where the loadings were not as expected, the factor analysis was re‐run following discussion with the research team and based on relevant theory and factor loadings (i.e. loadings <0.3 were considered low and those items were dropped).

For example, factor analysis with the community member data suggested that items assessing perceived stigma in community and healthcare settings were highly correlated and could be combined into one measure. However as in other studies, we wished to explore these domains of stigma separately. For example, recent research in The Gambia found that stigma experienced by PLHIV in healthcare settings led to more negative outcomes (e.g. delay in accessing treatment, poor adherence, etc.) than stigma experienced in the community, which had little or no impact on health outcomes 49. Similarly, studies in the US have found associations between anticipated stigma in the healthcare setting and poor adherence 52, mediated by internalized stigma and depressive symptoms 53. Therefore, we chose to keep these two aspects of perceived stigma separate (Table 1). The same process was undertaken with samples stratified by country, sex and type of HW (for HW items only) to determine if any differences in the results existed across these characteristics among the three study populations (Tables S1‐S3). In addition, factor analysis was undertaken, and factor loadings were obtained separately for each stigma domain using study samples where participants responded to all stigma items within a specific domain (Figure 1a‐c). 

**Table 1:** Factor loadings for original and re‐specified groupings from factor analyses for community members participating in the HPTN 071 (PopART) trial in South Africa and Zambia

Scale items	One factor		Two factors				Three factors					
	Original (n = 4821)	Re‐specified (n = 5060)	Original (n = 4821)		Re‐specified (n = 5060)		Original (n = 4821)			Re‐specified (n = 5060)		
	Factor 1^a^	‘Overall stigma’	Factor 1	Factor 2	Fear & judgement	Combined perceived stigma	Factor 1	Factor 2	Factor 3	Fear & judgement	Perceived stigma	
											Community	Healthcare settings
Domain: Fear & judgement												
I fear that I could contract HIV if I come into contact with the saliva of a person living with HIV	0.545	0.535	0.666	−0.070	0.599	0.104	0.777	0.039	−0.077	0.652	0.154	−0.049
I would not like to sit close to someone living with HIV, for example on public transport, at church or in a waiting room	0.544	0.549	0.813	−0.213	0.923	−0.092	0.813	−0.140	0.065	0.898	−0.045	−0.026
I would be ashamed if someone in my family had HIV	0.546	0.543	0.686	−0.089	0.468	0.203	0.446	−0.068	0.284	0.425	−0.067	0.300
Cronbach's alpha^b^			0.747		0.742		0.747			0.742		
Domain: Perceived stigma in the community												
People sometimes talk badly about people thought to be living with HIV to others	0.678	0.619	−0.110	0.941	−0.171	0.780	−0.120	0.898	0.051	−0.109	0.522	0.313
People thought to be living with HIV lose respect and standing	0.754	0.751	0.464	0.367	0.068	0.716	0.086	0.266	0.497	0.040	0.157	0.617
People thought to be living with HIV are verbally insulted, harassed and/or threatened	0.764	0.779	0.527	0.312	0.026	0.780	−0.033	0.129	0.751	−0.064	0.020	0.854
People thought to be living with HIV are sometimes physically assaulted	0.703	0.729	0.647	0.122	0.169	0.611	0.026	−0.099	0.859	0.052	−0.158	0.862
People sometimes disclose that other people are HIV positive without their permission	0.662	0.623	0.148	0.609	−0.062	0.691	0.117	0.634	0.043	0.025	0.962	−0.102
Cronbach's alpha											0.843	
Domain: Perceived stigma in healthcare settings												
Health workers sometimes talk badly about people living with or thought to be living with HIV to others	0.679	0.694	0.439	0.309	0.040	0.679	−0.034	0.158	0.627	0.003	0.093	0.639
Health workers sometimes disclose that other people are HIV positive without their permission	0.649	0.673	0.515	0.196	0.123	0.592	0.134	0.109	0.489	0.124	0.235	0.405
Cronbach's alpha						0.873^c^			0.758^d^			0.763
Items dropped												
People are hesitant to take an HIV test due to fear of other people's reaction if the test result is positive	0.620	–	−0.071	0.815	–	–	−0.058	0.808	0.005	–	–	–
PLHIV who are taking ART are treated better by others than PLHIV who are not taking ART	0.529	–	0.241	0.351		–	0.300	0.438	−0.081	–	–	–
Cronbach's alpha	0.892	0.879		0.884^e^				0.863^f^				

a Factor loadings were calculated based on the total sample used for the original and re‐specified factor analyses

b Cronbach's alphas were calculated in the same sample used for the factor analyses

c for all stigma items except those items in the fear & judgement domain and those items that were dropped

d perceived stigma (health setting) item

e for all stigma items except those items in the fear & judgement domain

f for all stigma items except those items in the fear & judgement domain and the perceived stigma (health setting) domain.

In the third phase, we calculated Cronbach's alpha for items grouped together under our domains of interest to assess the level of inter‐item agreement (i.e. reliability). This was done using complete data for each stigma domain (Figure 1a‐c) and complete data for CMs not living with HIV (Table 1). We considered an alpha value of 0.60 to 0.69 as “acceptable,” an alpha of 0.70 to 0.79 as “good” and an alpha of 0.80 or higher as “very good,” as suggested by Nunnally 54. Simple scales were calculated for each domain of stigma by summing the scores across the set of items in each domain and dividing by the total number of items. This method allowed each item to contribute the same amount to the overall score. Scores ranged between 0 and 3 with higher scores indicating greater levels of stigma. For the measures of reported experienced stigma, individual items were dichotomized (never = 0 vs. ever = 1) then collapsed to create two binary variables reflecting having experienced stigma in the community in the previous 12 months and having experienced stigma in the healthcare setting in the previous 12 months.

Last, we assessed construct validity by examining bivariate associations between the scales and variables previously reported as associated with the stigma domains these scales are intended to represent in the literature 47, including sex, age, education and ever having been tested for HIV (for CMs and HWs only) 14, 55. Women, younger participants and less‐educated participants were expected to report more internalized stigma 23, 56, 57, 58, 59. Among CMs and HWs, participants who were older, more educated and had tested for HIV were expected to report less negative attitudes towards PLHIV and less perceived stigma 14, 60, 61. Men were expected to perceive more stigma in the community and healthcare settings, given the difficulty engaging men in HIV care in sub‐Saharan Africa 62, 63. We used linear regression and performed Wald tests to assess associations with sex, age, education and ever tested for HIV on stigma domains adjusting for triplet to take into account the study design 39. All statistical analyses were conducted using the Stata software package (version 14.0, StataCorp LP, College Station, Texas).

### 2.4 Ethical considerations

Prior ethical approval for all study procedures was obtained from the institutional review boards of the London School of Hygiene and Tropical Medicine (LSHTM), Stellenbosch University, and the University of Zambia. All participants provided written informed consent prior to enrolment.

## 3 Results

### 3.1 Participants

Participants' characteristics are shown in Table 2. Among PLHIV, more participants were women (N = 3576/88%), aged 25 to 34 (N = 1874/46%) and unmarried (N = 2097/52%). Most PLHIV had completed secondary education (N = 2478/61%). Among community participants, more were women (N = 3826/66%) than men and between the ages of 18 and 24 (N = 2650/46%) than any other age category. Most community participants were unmarried (N = 3485/60%), had completed secondary education (N = 4087/71%), and reported ever having tested for HIV (N = 4851/84%). More HWs were Zambian (N = 999/64%), women (N = 1135/73%) and between the ages of 25 and 34 (N = 591/38%). Slightly more than half were married (N = 813/52%) and had completed further than secondary education (N = 822/53%). Almost all HWs (N = 1508/97%) reported ever having tested for HIV.

**Table 2:** Sociodemographic characteristics and stigma measures among people living with HIV, community members and health workers in South Africa and Zambia

Sociodemographic characteristics	People living with HIV N = 4053 N (%)	Community members N = 5782 N (%)	Health workers N = 1560 N (%)
Country			
Zambia	2285 (56.4)	3068 (53.1)	999 (64.0)
South Africa	1768 (43.6)	2714 (46.9)	561 (36.0)
Type of HW			
HFS	–	–	829 (53.1)
CHiP	–	–	508 (32.6)
CHW	–	–	223 (14.3)
Gender			
Male	477 (11.8)	1956 (33.8)	425 (27.2)
Female	3576 (88.2)	3826 (66.2)	1135 (72.8)
Age			
18 to 24	450 (11.1)	2650 (45.8)	163 (10.4)
25 to 34	1874 (46.2)	2082 (36.0)	591 (37.9)
35 to 44	1729 (42.7)	1050 (18.2)	370 (23.7)
45+	–	–	436 (27.9)
Marital status			
Not married	2097 (51.7)	3485 (60.3)	747 (47.9)
Married	1956 (48.3)	2297 (39.7)	813 (52.1)
Education			
Less than secondary	1416 (34.9)	1321 (22.8)	75 (4.8)
Completed secondary	2478 (61.1)	4087 (70.7)	663 (42.5)
Further	159 (3.9)	374 (6.5)	822 (52.7)
Ever tested for HIV			
No	–	931 (16.1)	52 (3.3)
Yes	–	4851 (83.9)	1508 (96.7)

Stigma scales and measures (% agreement with any statements)	n/N (%)	n/N (%)	n/N (%)
Fear and judgement	–	1227/5415 (22.7)	618/1362 (45.4)
Internalized stigma	887/3933 (22.6)	–	–
			
Perceived stigma in community	–	3338/5256 (63.5)	1306/1405 (93.0)
Experienced stigma in the community	868/3954 (22.0)	–	–
Perceived stigma in the healthcare setting (CMs)/perceived co‐worker stigma (HWs)	–	1607/5306 (30.3)	627/1445 (43.4)
Experienced stigma in healthcare settings	284/3974 (7.1)	–	–

CHiP, community HIV care providers; CHW, community‐based health worker delivering HIV‐related services; HFS, health facility‐based staff.

### 3.2 Factor analysis and reliability testing

Among PLHIV, three internalized stigma items were assessed using factor analysis. The items grouped together on one factor. The scale had high reliability in both the main (α = 0.816; Figure 1a) and sub‐group analyses by country and sex (Table S3).

Among CMs, 10 items were assessed. In initial analyses, the items grouped on one factor, an “overall stigma” scale (α = 0.879, Table 1) that would not allow us to examine the “fear and shame” and “perceived stigma” domains separately, as recommended by current stigma theory and intervention research 37, and would make comparisons of these domains across the three populations challenging. Therefore we examined the data using two factors, and found that items grouped onto a factor capturing fear and judgement (three items: α = 0.742, Table 1) and a factor capturing perceived stigma in community and healthcare settings (seven items, α = 0.873, Table 1).

However, based on previous research in the Gambia mentioned above, we determined that it was important to capture perceptions of stigma in community and healthcare settings distinctly 49. We also wanted to be able to compare CMs' perceptions with actual experiences of PLHIV in these two contexts. Thus we created two perceived stigma scales. One to assess stigma perceived in the community and the other to assess stigma perceived in the healthcare setting. Item agreement for the three community member scales was generally quite high: fear and judgement (three items, α = 0.742, Table 1), perceptions of stigma in the community (five items, α = 0.843, Table 1) and perceptions of stigma in the healthcare setting (two items, α = 0.763, Table 1). Sub‐group factor analysis by country and sex also supported high reliability in these domains (Table S1).

Among HWs, 14 items were assessed. In factor analysis, these items grouped into three separate factors with acceptable reliability representing fear and judgement (five items, α = 0.618; Figure 1c), perceived stigma in the community (five items, α = 0.647; Figure 1c) and perceived co‐worker stigma (four items, α = 0.769; Figure 1c). Sub‐group factor analysis confirmed acceptable reliability for all three scales by country, sex and type of health worker. However, Cronbach's alpha was below 0.6 for the fear and judgement domain and the perceived stigma in the community domain among CHWs and CHiPs (Table S2).

### 3.3 Scales and experience measures

We ultimately created seven scales. Mean scores for each scale are shown in Tables 3a, 3b, 3ca‐c. Means equal to or less than 1.0 indicate that on average, participants disagreed with the attitudinal statements. For PLHIV, the internalized stigma scale had a mean of 0.84 (standard deviation (SD) = 0.63) (Table 3a), with 22.6% reporting any internalized stigma in the past 12 months (Table 2). Experienced stigma and discrimination in the community and healthcare settings in the past 12 months were reported by 22.0% and 7.1% of PLHIV respectively (Table 2).

**Table 3a:** Internalised stigma, experienced stigma and discrimination, and associations with key sociodemographic characteristics for people living with HIV

Variables	Internalised stigma (n = 3933)			Stigma experienced in the community (n = 3954)			Stigma experienced In healthcare settings (n = 3974)			Any experienced stigma (n = 3947)		
	Mean score (SD)	Beta (95% CI)^a^	*p*_w_	n (%)	OR (95% CI)^b^	*p*_w_	n (%)	OR (95% CI)^b^	*p*_w_	n (%)	OR (95% CI)^b^	*p*_w_
Sex			0.631			0.161			0.075			0.090
Male	0.85 (0.65)	–		93 (19.8%)	1.00		23 (4.9%)	1.00		95 (20.3%)	1.00	
Female	0.84 (0.63)	−0.02 (−0.08 to 0.05)		775 (22.2%)	1.19 (0.93 to 1.52)		261 (7.4%)	1.49 (0.96 to 2.32)		814 (23.4%)	1.23 (0.97 to 1.57)	
Age group			**0.048**			**0.004**			**0.027**			**0.002**
18 to 24	0.89 (0.64)	–		78 (17.9%)	1.00		17 (3.9%)	1.00		79 (18.2%)	1.00	
25 to 34	0.86 (0.64)	−0.03 (−0.10 to 0.04)		377 (20.6%)	1.23 (0.94 to 1.62)		133 (7.2%)	1.87 (1.11 to 3.14)		397 (21.8%)	1.29 (0.99 to 1.69)	
35 to 44	0.81 (0.63)	−0.07 (−0.14 to − 0.00)		413 (24.4%)	1.50 (1.14 to 1.97)		134 (7.9%)	2.04 (1.21 to 3.42)		433 (25.6%)	1.57 (1.20 to 2.05)	
Education			0.061			**0.046**			0.843			**0.049**
Less than secondary	0.84 (0.65)	–		334 (24.2%)	1.00		80 (5.8%)	1.00		349 (25.4%)	1.00	
Completed secondary	0.85 (0.63)	−0.02 (−0.07 to 0.02)		490 (20.3%)	0.81 (0.68 to 0.96)		193 (7.9%)	1.04 (0.77 to 1.40)		515 (21.3%)	0.81 (0.68 to 0.96)	
Post secondary	0.72 (0.61)	−0.13 (−0.24 to − 0.02)		44 (28%)	0.99 (0.68 to 1.46)		11 (7%)	0.87 (0.44 to 1.69)		45 (28.7%)	0.95 (0.65 to 1.40)	
Overall – Mean score (SD)^c^	0.84 (0.63)											

The bolded values reflect statistically significant *p* values (values < 0.05).

CI, confidence Interval; OR: odds ratio; *p_w_*, *p* value for the Wald test; SD, standard deviation.

a Beta with 95% confidence interval obtained from linear regression

b odds ratios with 95% confidence interval obtained from logistic regression

c summary scores range from 0 to 3, with higher scores reflecting more stigmatizing responses.

**Table 3b:** Mean scores for each stigma scale and associations with key sociodemographic characteristics for community members

Variables	Fear and judgement (n = 5415)			Perceived stigma in the community (n = 5256)			Perceived stigma in healthcare settings (n = 5306)			
	Mean score (SD)	Beta (95% CI)^a^	*p*_w_	Mean score (SD)	Beta (95% CI)^a^	*p*_w_	Mean score (SD)	Beta (95% CI)^a^	*p*_w_	
Sex			0.110			0.103			**0.006**	
Male	0.83 (0.59)	–		1.26 (0.66)	–		1.07 (0.75)	–		
Female	0.80 (0.58)	−0.03 (−0.06 to 0.01)		1.23 (0.66)	−0.03 (−0.07 to 0.01)		0.99 (0.73)	−0.06 (−0.10 to − 0.02)		
Age group			0.184			0.862			0.745	
18 to 24	0.82 (0.59)	–		1.24 (0.66)	–		1.00 (0.73)	–		
25 to 34	0.80 (0.57)	−0.03 (−0.06 to 0.00)		1.23 (0.66)	0.00 (−.04 to 0.04)		1.02 (0.73)	−0.01 (−0.05 to 0.04)		
35 to 44	0.80 (0.59)	−0.03 (−0.07 to 0.02)		1.24 (0.67)	0.01 (−0.03 to 0.06)		1.05 (0.77)	0.02 (−0.04 to 0.07)		
Education			0.539			**<0.001**			**<0.001**	
Less than secondary	0.79 (0.58)	–		1.20 (0.66)	–		0.89 (0.73)	–		
Completed secondary	0.81 (0.58)	−0.02 (−0.06 to 0.02)		1.23 (0.66)	0.02 (−0.02 to 0.07)		1.04 (0.73)	0.06 (0.01 to 0.11)		
Post secondary	0.85 (0.64)	0.00 (−0.07 to 0.07)		1.42 (0.66)	0.18 (0.10 to 0.26)		1.20 (0.79)	0.20 (0.11 to 0.29)		
Ever tested for HIV			0.449			**<0.001**			0.191	
No	0.78 (0.63)	–		1.14 (0.74)	–		1.00 (0.81)	–		
Yes	0.82 (0.57)	0.02 (−0.03 to 0.06)		1.25 (0.64)	0.09 (0.04 to 0.14)		1.02 (0.72)	0.04 (−0.02 to 0.09)		
Overall to Mean score (SD)^b^	0.81 (0.58)			1.24 (0.66)			1.02 (0.74)			

The bolded values reflect statistically significant *p* values (values < 0.05).

CI, confidence Interval; *p_w_*, *p* value for the Wald test; SD, Standard deviation.

a Beta with 95% confidence interval obtained from linear regression

b summary scores range from 0 to 3, with higher scores reflecting more stigmatizing responses.

**Table 3c:** Mean scores for each stigma scale and associations with key sociodemographic characteristics among health workers

	Fear and judgement (n = 1362)			Perceived stigma in the community (n = 1405)			Perceived co‐worker stigma (n = 1445)		
Variables	Mean score (SD)	Beta (95% CI)^a^	*p*_w_	Mean score (SD)	Beta (95% CI)^a^	*p*_w_	Mean score (SD)	Beta (95% CI)^a^	*p*_w_
Sex			0.253			**0.019**			**0.022**
Male	0.80 (0.50)	–		1.52 (0.50)	–		1.01 (0.62)	–	
Female	0.77 (0.49)	−0.03 (−0.09 to 0.02)		1.49 (0.51)	−0.07 (−0.13 to − 0.01)		0.89 (0.60)	−0.08 (−0.15 to − 0.01)	
Age group			**0.015**			**0.027**			**0.019**
18 to 24	0.81 (0.48)	–		1.60 (0.41)	–		0.85 (0.59)	–	
25 to 34	0.73 (0.46)	−0.07 (−0.16 to 0.02)		1.53 (0.50)	−0.04 (−0.14 to 0.05)		0.86 (0.60)	0.02 (−0.09 to 0.13)	
35 to 44	0.78 (0.48)	−0.02 (−0.12 to 0.08)		1.50 (0.51)	−0.08 (−0.18 to 0.02)		0.98 (0.58)	0.13 (0.02 to 0.25)	
45+	0.84 (0.53)	0.04 (−0.06 to 0.13)		1.43 (0.53)	−0.13 (−0.23 to − 0.03)		0.98 (0.63)	0.09 (−0.02 to 0.20)	
Education			**<0.001**			**<0.001**			**0.012**
Less than secondary	0.92 (0.66)	–		1.31 (0.60)	–		0.87 (0.59)	–	
Completed secondary	0.87 (0.50)	−0.03 (−0.15 to 0.10)		1.59 (0.49)	0.23 (0.10 to 0.35)		0.94 (0.63)	0.16 (0.01 to 0.31)	
Post secondary	0.70 (0.45)	−0.21 (−0.33 to − 0.09)		1.45 (0.50)	0.12 (−0.00 to 0.24)		0.91 (0.59)	0.07 (−0.08 to 0.22)	
Ever tested for HIV			**0.002**			0.912			0.192
No	1.02 (0.58)	–		1.52 (0.61)	–		1.04 (0.52)	–	
Yes	0.77 (0.49)	−0.24 (−0.39 to − 0.09)		1.50 (0.50)	−0.01 (−0.16 to 0.14)		0.92 (0.61)	−0.11 (−0.29 to 0.06)	
Overall – Mean score (SD)^b^	0.78 (0.49)			1.50 (0.51)			0.92 (0.60)		

The bolded values reflect statistically signficant *p* values (values < 0.05).

CI, confidence interval; *p_w_*, *p* value for the Wald test; SD, Standard deviation.

a Beta with 95% confidence interval obtained from linear regression

b summary scores range from 0 to 3, with higher scores reflecting more stigmatizing responses.

For CMs, the mean score was 0.81 (SD = 0.58) for HIV fear and judgement, with 22.7% agreeing with at least one of the three statements, 1.24 (SD = 0.66) for perceived stigma in the community, with 63.5% agreeing with at least one of the five statements, and 1.02 (SD = 0.74) for perceived stigma in the healthcare setting, with 30.3% agreeing with at least one of the two statements (Tables 2 and 3b).

Among HWs, the mean score was 0.78 (SD = 0.49) for fear and judgement, with 45.4% agreeing with any of the five statements, 1.50 (SD = 0.51) for perceived stigma in the community, with 93.0% agreeing with any of the five statements, and 0.92 (SD = 0.60) for perceived co‐worker stigma, with 43.4% agreeing with any of the four statements (Tables 2 and 3c).

### 3.4 Stigma and sociodemographic factors

As expected, internalized stigma was associated with age among PLHIV. Older participants reported slightly less internalized stigma (aged 35 to 44 years, mean = 0.81; SD = 0.63) than younger participants (aged 18 to 24 years, mean = 0.89; SD = 0.64; *p* = 0.048). PLHIV aged 35 to 44 were more likely to report experiencing stigma and discrimination in the community than PLHIV aged 18 to 24 (OR: 1.50; 95% CI: 1.14 to 1.97). PLHIV who completed secondary school were less likely than PLHIV with less than secondary education to report experienced stigma in the community (OR: 0.81; 95% CI: 0.68 to 0.96). While mean internalized stigma scores were higher for PLHIV with less education, the observed difference was not significant. Unexpectedly, internalized stigma was not associated with sex. Experienced stigma in the healthcare setting was more likely to be reported by PLHIV aged 25 to 34 (OR: 1.87; 95% CI: 1.11 to 3.14) and those aged 35 to 44 (OR: 2.04; 95% CI: 1.21 to 3.42) compared to younger PLHIV aged 18 to 24 years. Experienced stigma did not differ by sex (Table 3a).

Contrary to our hypotheses, HIV fear and judgement did not vary significantly by sex, age, education or ever tested for HIV among CMs. Perceptions of stigma in the community were not associated with sex, but were significantly associated with education and ever tested for HIV in the opposite direction as expected. Unexpectedly, those with post‐secondary education (mean = 1.42; SD = 0.66; *p* < 0.001) and those who had ever tested for HIV (mean = 1.25; SD = 0.64; *p* < 0.001) perceived more stigma in the community than those with less than secondary education and those who had not tested for HIV respectively. Perceived stigma in the healthcare setting was associated with both sex and education. As expected, men (mean = 1.07; SD = 0.75; *p* = 0.006) perceived more stigma in healthcare settings than women. Unexpectedly, those with secondary (mean = 1.04; SD = 0.73; *p* < 0.001) or post‐secondary education (mean = 1.20; SD = 0.79; *p* < 0.001) perceived more stigma in the healthcare setting than those with less than secondary education (Table 3b).

Among HWs, fear and judgement were significantly associated with age, education and ever having tested for HIV. Contrary to our hypotheses, younger (aged 18 to 24, mean = 0.81; SD = 0.48; *p* = 0.015) and older (aged 45 + years, mean = 0.84; SD = 0.53, *p* = 0.015) HWs had higher scores than HWs aged 25 to 44 years, reflecting more negative attitudes. As expected, HWs with less than secondary education (mean = 0.92; SD = 0.66; *p* < 0.001) and those who had never tested for HIV (mean = 1.02; SD = 0.58; *p* = 0.002) reported more negative attitudes. Perceived stigma in the community was significantly associated with sex, age and education. As expected, male HWs (mean = 1.52; SD = 0.50; *p* = 0.019) and younger HWs aged 18 to 24 (mean = 1.60; SD = 0.41; *p* = 0.027) perceived more stigma in the community. Unexpectedly, HWs who had completed secondary education (mean = 1.59; SD = 0.49; *p* < 0.001) perceived more stigma in the community and perceived more co‐worker stigma in the healthcare setting (mean = 0.94; SD = 0.63; *p* = 0.012). Perceived co‐worker stigma was significantly associated with sex and age, with male HWs (mean = 1.01; SD = 0.62; *p* = 0.022), and those over 35 perceiving more co‐worker stigma (35 to 44: mean = 0.98; SD = 0.58; *p* = 0.019; 45+: mean = 0.98; SD = 0.63). Ever tested for HIV was not associated with perceived stigma in the community or perceived co‐worker stigma for HWs (Table 3c).

## 4 Discussion

We developed seven scales and two experience measures using 35 items capturing drivers and manifestations of HIV stigma and discrimination and established their reliability and validity across three populations in Zambia and South Africa. The health worker scales are applicable in both countries and with a broad range of HWs. Our study is the first to create theory‐based, parallel measures of stigma across PLHIV, CMs and HWs in the same study population. More CMs and HWs reported perceived stigma in community and healthcare settings than the proportion of PLHIV who reported experiences of stigma in both settings. Men perceived more stigma towards PLHIV in healthcare settings, which may be a factor influencing male engagement in HIV services.

A few limitations in our approach should be mentioned. First, we did not examine predictive validity, as we only included baseline data in the present analyses. Future analyses are planned to assess how well our parallel measures of stigma predict relevant outcomes such as ART adherence among PLHIV. Second, while the parallel measures developed allow for quantitative assessment of stigma, it should be noted that stigma and discrimination emerge from a social process influenced by power and inequality 64. Qualitative social science investigations that aim to understand the social dimensions of stigma, such as those conducted in the HPTN 071 (PopART) trial, will complement the scales measured here by describing how stigma is influencing and being influenced by social and inter‐personal processes 65. Third, men were underrepresented in the population cohort (PC) of the HPTN 071 (PopART) trial 66, despite repeat visits to households, additional recruitment efforts added, and continuous community engagement efforts 67. The higher level of perceived stigma in the healthcare setting reported by men in the present study suggests that stigma may have discouraged men's participation in the trial. Given this, it is possible that the prevalence of the various stigma domains presented in the present paper across our three populations may be underestimates, especially for men in the study communities. Lastly, assessing stigma quantitatively among members of key populations in the PC was not within the scope of our study. Thus, we were not able to assess intersectional stigma nor validate our stigma measures among key populations living with HIV. Key populations were included in our social science investigations and analyses of these data are forthcoming. Despite these limitations, our study draws strength from being the first to develop and validate parallel measures of stigma across three populations in the context of a large HIV prevention trial, including the first ever population‐based, random sample of PLHIV.

Our resulting scales measured different domains of stigma than the parallel scales developed by Visser et al. 13. For CMs and HWs, our “fear and judgement” scales combine some of the items assessing “blame and judgement” and “interpersonal distancing” with items assessing shame and fear of HIV infection through casual contact with PLHIV. Our parallel measures for CMs and HWs also differed in that they included two new scales to assess perceptions of stigma occurring in a community or healthcare setting, which previous research suggests may prevent or delay engagement with health services 49. Like Visser, we created a scale to capture internalized, or “self‐stigma,” among PLHIV. Similar to previous research in Bangladesh and South Africa, we found that older age was associated with lower levels of internalized stigma 24, 68. In contrast to Visser's scale of expected stigma, we developed two composite measures that capture reported experiences of stigma and discrimination in community and healthcare settings over the past 12 months. This enabled us to assess the baseline prevalence and characteristics of two critical domains of HIV stigma that have been shown to be associated with HIV‐related health seeking behaviour among PLHIV 49, 69.

Similar to Visser et al. and Zelaya et al., the parallel scales among the community and PLHIV samples showed good to very good internal consistency 13, 26. Among the complete healthcare worker sample, the internal consistency of the “fear and judgement” scale and “perceived stigma in the community” scales was lower than desired, yet acceptable for making group comparisons (α > 0.60) 54. The findings from the sub‐group analyses of the stigma scales among the three different cadres of HWs suggest that some items may be more appropriate for facility‐based staff. For example, two items on the “fear and judgement” scale (“Other people deserve access to health services more than PLHIV” and “I fear that I could contract HIV if I come into contact with the saliva of a person living with HIV”) had low factor loadings for CHiPs only. These items may not resonate with CHiPs, who have chosen to provide door‐to‐door HIV testing, counselling and referral services for PLHIV in the community and have been trained on transmission of HIV and provision of culturally sensitive services. The items we used in this research were adapted from a facility‐based stigma survey 33. As the factor analyses of the other two scales (i.e. perceived stigma in the community and perceived co‐worker stigma) were comparable in the combined health worker sample and the sub‐group analyses, we are comfortable recommending the utility of the three scales in combined analyses with the health worker population. However, future stigma research could consider alternative items that may be more relevant for assessing the drivers of stigma in the context of community‐based HIV‐related service provision.

Similar to a multi‐country study conducted among HWs 33, fear and judgement among CMs and HWs in our study population were low, reflecting little fear, shame and judgement towards PLHIV. These low levels may explain why we did not see any differences in negative attitudes across socio‐demographic characteristics and suggests that interventions to reduce negative attitudes towards PLHIV may not need to be targeted to specific demographic groups. Conversely, perceptions of stigma in the community and healthcare settings were high for both groups, with HWs perceiving considerably more stigma occurring in the community setting. We were not able to compare our findings with other studies due to the paucity of published data on this aspect of stigma among CMs and HWs. However, we do know that in our study population, the proportions of PLHIV reporting internalized and experienced stigma were significantly associated with the level of perceived stigma in the community reported by CMs, but not with the level reported by HWs 70. This finding resonates with research in the US that found potential causal pathways among these different dimensions of stigma, in particular that women living with HIV who perceive high levels of stigma in the community and in healthcare settings are more likely to internalize stigma 53. Our findings that men and more educated CMs and HWs perceived more stigma suggest that interventions to reduce perceived stigma may need to be targeted to specific demographic groups. Further research is warranted to better understand how perceived stigma is operating in communities and healthcare settings, why HWs might perceive more stigma than CMs, and how high levels of perceived stigma may impact health outcomes for PLHIV.

Nearly one in four PLHIV reported internalized or experienced stigma in the last year, and the levels of stigma did not differ by sex, suggesting that interventions to reduce these key stigma manifestations may be needed for both men and women. While reports of experienced stigma in the healthcare setting were relatively low, previous research suggests that such experiences can be particularly damaging and impactful for PLHIV, and anticipation of stigma in this setting can have significant impacts on HIV prevention and treatment utilization 71. Comparison of the parallel measures developed does suggest some unique domains of stigma for each population that may require specific intervention. For example, stigma reduction efforts for young PLHIV should likely focus on addressing and overcoming internalized stigma, whereas older PLHIV may require support to cope with stigma experienced in the community and healthcare settings.

Likewise, efforts with HWs could emphasize the high levels of perceived stigma in the community compared to actual experiences of stigma reported by PLHIV, which may help HWs living with HIV to feel more comfortable disclosing their status and accessing care at the facilities and communities in which they work 72. Our finding that men perceived more stigma towards PLHIV in the healthcare setting than women could partially explain why engaging men in HIV‐related health services has been challenging in sub‐Saharan Africa to date 73, suggesting that interventions to address perceptions about HIV stigma in communities may be needed to enhance male engagement in the HIV prevention and treatment cascades. Having access to parallel data on stigma domains that can be influenced by interventions could be very useful for policy makers and programme planners as they determine where to direct limited financial resources for national‐level HIV responses.

## 5 Conclusions

The successful development of parallel scales in this study will enable future research to answer a number of key questions for the delivery of HIV services, such as whether internalized stigma is a greater barrier to ART uptake and engagement in care among PLHIV than experienced stigma, and to compare the impact of different types of stigma on the uptake of HIV prevention and care services longitudinally. Knowing the answers to these questions could then guide efforts to address the most pressing domains of stigma and in turn remove barriers to HIV prevention, care and treatment.

## Competing interest

None declared.

## Authors' Contributions

AS, JH, VB and GH conceptualized the manuscript. PL, TP and MS conducted the analysis. HM, TM and SK oversaw in‐country data collection. NB‐M, RV, ASh and DD managed the PC data and HM, SK and TP managed the health worker data sets. AS led the manuscript writing. KS conducted the literature review. JH, GH, VB, TM, HM, SK, TP, PL, K, MS, DD, PB, HA and RH were involved in drafting the manuscript and provided critical feedback on the full manuscript. All authors read and approved the final manuscript.

## Supporting information


**Figure S1**. Flowcharts for community members, health workers and people living with HIV.

**Table S1**. Factor loadings from factor analyses for community members participating in the PopART (HPTN 071) trial in South Africa and Zambia, by country and sex
**Table S2**. Factor loadings from factor analyses for health workers participating in the PopART (HPTN 071) trial in South Africa and Zambia, by country, sex and type of health worker
**Table S3**. Factor loadings from factor analyses for PLHIV participating in the PopART (HPTN 071) trial in South Africa and Zambia, by country and sex.

## References

[1] Cowan FM , Delany‐Moretlwe S , Sanders EJ , Mugo NR , Guedou FA , Alary M , et al. PrEP implementation research in Africa: what is new? J Int AIDS Soc. 2016;19(7(Suppl 6)):21101. 27760680 10.7448/IAS.19.7.21101 PMC5071780

[2] Weiss SM , Zulu R , Jones DL , Redding CA , Cook R , Chitalu N . The Spear and Shield intervention to increase the availability and acceptability of voluntary medical male circumcision in Zambia: a cluster randomised controlled trial. Lancet HIV. 2015;2(5):e181–9. 26120594 10.1016/S2352-3018(15)00042-9 PMC4478609

[3] Cohen MS , Chen YQ , McCauley M , Gamble T , Hosseinipour MC , Kumarasamy N , et al. Antiretroviral Therapy for the Prevention of HIV‐1 Transmission. N Engl J Med. 2016;375(9):830–9. 27424812 10.1056/NEJMoa1600693 PMC5049503

[4] Amico KR , Mansoor LE , Corneli A , Torjesen K , van der Straten A . Adherence Support Approaches in Biomedical HIV Prevention Trials: Experiences, Insights and Future Directions from Four Multisite Prevention Trials. AIDS Behav. 2013;17(6):2143–55. 23435697 10.1007/s10461-013-0429-9 PMC3672509

[5] Stirratt MJ , Gordon CM . Adherence to biomedical HIV prevention methods: Considerations drawn from HIV treatment adherence research. Curr HIV/AIDS Rep. 2008;5(4):186–92. 18838058 10.1007/s11904-008-0027-z

[6] Gourlay A , Birdthistle I , Mburu G , Iorpenda K , Wringe A . Barriers and facilitating factors to the uptake of antiretroviral drugs for prevention of mother‐to‐child transmission of HIV in sub‐Saharan Africa: a systematic review. J Int AIDS Soc. 2013;16(1):18588. 23870277 10.7448/IAS.16.1.18588 PMC3717402

[7] Mahajan AP , Sayles JN , Patel VA , Remien RH , Sawires SR , Ortiz DJ , et al. Stigma in the HIV/AIDS epidemic: a review of the literature and recommendations for the way forward. AIDS. 2008;22(Suppl 2):S67–79. 10.1097/01.aids.0000327438.13291.62 PMC2835402 18641472

[8] Marrazzo JM , Ramjee G , Richardson BA , Gomez K , Mgodi N , Nair G , et al. Tenofovir‐based preexposure prophylaxis for HIV infection among African women. N Engl J Med. 2015;372(6):509–18. 25651245 10.1056/NEJMoa1402269 PMC4341965

[9] Van Damme L , Corneli A , Ahmed K , Agot K , Lombaard J , Kapiga S , et al. Preexposure prophylaxis for HIV infection among African Women. N Engl J Med. 2012;5367(2):411–22. 10.1056/NEJMoa1202614 PMC3687217 22784040

[10] Celum CL , Delany‐Moretlwe S , McConnell M , van Rooyen H , Bekker L‐G , Kurth A , et al. Rethinking HIV prevention to prepare for oral PrEP implementation for young African women. J Int AIDS Soc. 2015;18(4 (Suppl 3)):20227. 26198350 10.7448/IAS.18.4.20227 PMC4509892

[11] Pescosolido BA , Martin JK , Lang A , Olafsdottir S . Rethinking theoretical approaches to stigma: a Framework Integrating Normative Influences on Stigma (FINIS). Soc Sci Med. 2008;67(3):431–40. 18436358 10.1016/j.socscimed.2008.03.018 PMC2587424

[12] Nyblade LC . Measuring HIV stigma: existing knowledge and gaps. Psychol Health Med. 2006;11(3):335–45. 17130069 10.1080/13548500600595178

[13] Visser MJ , Kershaw T , Makin JD , Forsyth BWC . Development of parallel scales to measure HIV‐related stigma. AIDS Behav. 2008;12(5):759–71. 18266101 10.1007/s10461-008-9363-7 PMC4244074

[14] Kalichman SC , Simbayi LC , Jooste S , Toefy Y , Cain D , Cherry C , et al. Development of a brief scale to measure AIDS‐related stigma in South Africa. AIDS Behav. 2005;9(2):135–43. 15933833 10.1007/s10461-005-3895-x

[15] Beaulieu M , Adrien A , Potvin L , Dassa C . Comité consultatif sur les attitudes envers les PVVIH C consultatif sur les attitudes envers les. Stigmatizing attitudes towards people living with HIV/AIDS: validation of a measurement scale. BMC Public Health. 2014;4(14):1246. 10.1186/1471-2458-14-1246 PMC4289343 25476441

[16] Herek GM , Capitanio JP , Widaman KF . Stigma, social risk, and health policy: public attitudes toward HIV surveillance policies and the social construction of illness. Heal Psychol. 2003;22(5):533–40. 10.1037/0278-6133.22.5.533 14570537

[17] Herek GM , Capitanio JP , Widaman KF . HIV‐related stigma and knowledge in the United States: prevalence and trends, 1991–1999. Am J Public Health. 2002;92(3):371–7. 11867313 10.2105/ajph.92.3.371 PMC1447082

[18] Hamra M , Ross MW , Orrs M , D'Agostino A . Relationship between expressed HIV/AIDS‐related stigma and HIV‐beliefs/knowledge and behaviour in families of HIV infected children in Kenya. Trop Med Int Health. 2006;11(4):513–27. 16553935 10.1111/j.1365-3156.2006.01583.x

[19] Genberg BL , Kawichai S , Chingono A , Sendah M , Chariyalertsak S , Konda KA , et al. Assessing HIV/AIDS stigma and discrimination in developing countries. AIDS Behav. 2008;12(5):772–80. 18080830 10.1007/s10461-007-9340-6 PMC2745205

[20] Zelaya CE , Sivaram S , Johnson SC , Srikrishnan AK , Solomon S , Celentano DD . HIV/AIDS Stigma: reliability and validity of a new measurement instrument in Chennai. India. AIDS Behav. 2008;12(5):781–8. 18030613 10.1007/s10461-007-9331-7

[21] Berger BE , Ferrans CE , Lashley FR . Measuring stigma in people with HIV: psychometric assessment of the HIV stigma scale. Res Nurs Health. 2001;24(6):518–29. 11746080 10.1002/nur.10011

[22] Holzemer WL , Uys LR , Chirwa ML , Greeff M , Makoae LN , Kohi TW , et al. Validation of the HIV/AIDS Stigma instrument—PLWA (HASI‐P). AIDS Care. 2007;19(8):1002–12. 17851997 10.1080/09540120701245999

[23] Sayles JN , Hays RD , Sarkisian CA , Mahajan AP , Spritzer KL , Cunningham WE . Development and psychometric assessment of a multidimensional measure of internalized HIV Stigma in a sample of HIV‐positive adults. AIDS Behav. 2008;12(5):748–58. 18389363 10.1007/s10461-008-9375-3 PMC2858334

[24] Kalichman SC , Simbayi LC , Cloete A , Mthembu PP , Mkhonta RN , Ginindza T . Measuring AIDS stigmas in people living with HIV/AIDS: the Internalized AIDS‐Related Stigma Scale. AIDS Care. 2009;21(1):87–93. 19085224 10.1080/09540120802032627

[25] Phillips KD , Moneyham L , Tavakoli A . Development of an instrument to measure internalized Stigma in those with HIV/AIDS. Issues Ment Health Nurs. 2011;32(6):359–66. 21692574 10.3109/01612840.2011.575533

[26] Zelaya CE , Sivaram S , Johnson SC , Srikrishnan AK , Suniti S , Celentano DD . Measurement of self, experienced, and perceived HIV/AIDS stigma using parallel scales in Chennai, India. AIDS Care. 2012;24(7):846–55. 22272891 10.1080/09540121.2011.647674

[27] Kipp AM , Audet CM , Earnshaw VA , Owens J , McGowan CC , Wallston KA . Re‐validation of the Van Rie HIV/AIDS‐related stigma scale for use with people living with HIV in the United States. PLoS ONE. 2015;10(3):e0118836. 25738884 10.1371/journal.pone.0118836 PMC4349586

[28] Mahendra VS , Gilborn L , Bharat S , Mudoi R , Gupta I , George B , et al. Understanding and measuring AIDS‐related stigma in health care settings: a developing country perspective. SAHARA J. 2007;4(2):616–25. 18071613 10.1080/17290376.2007.9724883

[29] Stein JA , Li L . Measuring HIV‐related Stigma among chinese service providers: confirmatory factor analysis of a multidimensional scale. AIDS Behav. 2008;12(5):789–95. 18064554 10.1007/s10461-007-9339-z PMC2700338

[30] Varas‐Díaz N , Neilands TB . Development and validation of a culturally appropriate HIV/AIDS Stigma Scale for Puerto Rican health professionals in training. AIDS Care. 2009;21(10):1259–70. 20024702 10.1080/09540120902804297 PMC2802456

[31] Uys LR , Holzemer WL , Chirwa ML , Dlamini PS , Greeff M , Kohi TW , et al. The development and validation of the HIV/AIDS Stigma Instrument ‐ Nurse (HASI‐N). AIDS Care. 2009;21(2):150–9. 19229683 10.1080/09540120801982889 PMC2716132

[32] Rutledge SE , Whyte J , Abell N , Brown KM , Cesnales NI . Measuring Stigma among health care and social service providers: the hiv/aids provider stigma inventory. AIDS Patient Care STDS. 2011;25(11):673–82. 21967495 10.1089/apc.2011.0008

[33] Nyblade L , Jain A , Benkirane M , Li L , Lohiniva A‐L , McLean R , et al. A brief, standardized tool for measuring HIV‐related stigma among health facility staff: results of field testing in China, Dominica, Egypt, Kenya, Puerto Rico and St. Christopher & Nevis. J Int AIDS Soc. 2013;16(3 (Suppl 2)):18718. 24242266 10.7448/IAS.16.3.18718 PMC3833189

[34] Wagner AC , Hart TA , McShane KE , Margolese S , Girard TA . Health care provider attitudes and beliefs about people living with HIV: initial validation of the health care provider HIV/AIDS Stigma Scale (HPASS). AIDS Behav. 2014;18(12):2397–408. 24965675 10.1007/s10461-014-0834-8

[35] Modgill G , Patten SB , Knaak S , Kassam A , Szeto AC . Opening Minds Stigma Scale for Health Care Providers (OMS‐HC): examination of psychometric properties and responsiveness. BMC Psychiatry. 2014;14(1):120. 24758158 10.1186/1471-244X-14-120 PMC4024210

[36] Ahmadi K , Reidpath DD , Allotey P , Hassali MAA . A latent trait approach to measuring HIV/AIDS related stigma in healthcare professionals: application of mokken scaling technique. BMC Med Educ. 2016;16:155. 27240562 10.1186/s12909-016-0676-3 PMC4885119

[37] Stangl AL , Lloyd JK , Brady LM , Holland CE , Baral S . A systematic review of interventions to reduce HIV‐related stigma and discrimination from 2002 to 2013: how far have we come? J Int AIDS Soc. 2013;16(3 (Suppl 2)):18734. 24242268 10.7448/IAS.16.3.18734 PMC3833106

[38] Hargreaves J , Stangl A , Bond V , Hoddinott G , Krishnaratne S , Mathema H , et al. P14.14 Intersecting stigmas: a framework for data collection and analysis of stigmas faced by people living with hiv and key populations. Sex Transm Infect. 2015;91 Suppl 2:A203.

[39] Hayes R , Ayles H , Beyers N , Sabapathy K , Floyd S , Shanaube K , et al. HPTN 071 (PopART): Rationale and design of a cluster‐randomised trial of the population impact of an HIV combination prevention intervention including universal testing and treatment – a study protocol for a cluster randomised trial. Trials. 2014;15(1):57. 24524229 10.1186/1745-6215-15-57 PMC3929317

[40] Stangl AL , Brady L , Fritz K . Measuring HIV stigma and discrimination. Washington, DC: International Center for Research on Women, 2012; p. 1–2.

[41] Stangl AL , Earnshaw VA , Logie CH , Van Brakel W , Simbayi LC , Barré I , et al. The Health Stigma and Discrimination Framework: a global, crosscutting framework to inform research, intervention development, and policy on health‐related stigmas. BMC Med. 2019. 10.1186/s12916-019-1271-3 PMC6376797 30764826

[42] Rao D , Desmond M , Andrasik M , Rasberry T , Lambert N , Cohn SE , et al. Feasibility, acceptability, and preliminary efficacy of the unity workshop: an internalized stigma reduction intervention for African American women living with HIV. AIDS Patient Care STDS. 2012;26(10):614–20. 22984780 10.1089/apc.2012.0106 PMC3462391

[43] Kane JC , Elafros MA , Murray SM , Mitchell EMH , Augustinavicius JL , Causevic S , et al. A scoping review of health‐related stigma outcomes for high‐burden diseases in low‐ and middle‐income countries. BMC Med. 2019. 10.1186/s12916-019-1250-8 PMC6376728 30764819

[44] Earnshaw VA , Smith LR , Chaudoir SR , Lee I‐C , Copenhaver MM . Stereotypes about people living with HIV: implications for perceptions of hiv risk and testing frequency among at‐risk populations. AIDS Educ Prev. 2012;24(6):574–81. 23206205 10.1521/aeap.2012.24.6.574 PMC3641644

[45] Hargreaves JR , Krishnaratne S , Mathema H , Lilleston PS , Sievwright K , Mandla N , et al. Individual and community‐level risk factors for HIV stigma in 21 Zambian and South African communities: analysis of data from the HPTN071 (PopART) study. AIDS. 2018;32(6):783–793. 29369164 10.1097/QAD.0000000000001757 PMC5854764

[46] Hargreaves JR , Stangl A , Bond V , Hoddinott G , Krishnaratne S , Mathema H , et al. HIV‐related stigma and universal testing and treatment for HIV prevention and care: design of an implementation science evaluation nested in the HPTN 071 (PopART) cluster‐randomized trial in Zambia and South Africa. Health Policy Plan. 2016;31(10):1342–54. 27375126 10.1093/heapol/czw071 PMC6702767

[47] Cronbach LJ , Meehl P . Construct validity in psychological tests. Psychol Bull. 1955;52(4):281–301. 13245896 10.1037/h0040957

[48] dos Santos MM , Kruger P , Mellors SE , Wolvaardt G , van der Ryst E . An exploratory survey measuring stigma and discrimination experienced by people living with HIV/AIDS in South Africa: the People Living with HIV Stigma Index. BMC Public Health. 2014;14(1):80. 24461042 10.1186/1471-2458-14-80 PMC3909177

[49] Peitzmeier SM , Grosso A , Bowes A , Ceesay N , Baral SD . Associations of Stigma with negative health outcomes for people living with HIV in the Gambia. JAIDS J Acquir Immune Defic Syndr. 2015;68:S146–53. 25723979 10.1097/QAI.0000000000000453

[50] Fabrigar L , Wegener D , MacCallum RSE . Evaluating the use of exploratory factor analysis in psychological research. Psychol Methods. 1999;4(3):272–99.

[51] Fabrigar LR , Wegener DT . Exploratory factor analysis. Oxford: Oxford University Press; 2011.

[52] Rice WS , Turan B , Fletcher FE , Nápoles TM , Walcott M , Batchelder A , et al. A mixed methods study of anticipated and experienced stigma in health care settings among women living with HIV in the united states. AIDS Patient Care STDS. 2019;33(4):184–95. 30932700 10.1089/apc.2018.0282 PMC6459270

[53] Turan B , Rogers AJ , Rice WS , Atkins GC , Cohen MH , Wilson TE , et al. Association between perceived discrimination in healthcare settings and hiv medication adherence: mediating psychosocial mechanisms. AIDS Behav. 2017;21(12):3431–4339. 29081045 10.1007/s10461-017-1957-5 PMC5705383

[54] Nunnally JC . Assessment of reliability In: Psychometric theory. 2nd ed. New York: McGraw‐Hill; 1978 p. 245–6.

[55] Kalichman SC , Simbayi LC . HIV testing attitudes, AIDS stigma, and voluntary HIV counselling and testing in a black township in Cape Town. South Africa. Sex Transm Infect. 2003;79(6):442–7. 14663117 10.1136/sti.79.6.442 PMC1744787

[56] Carr RL , Gramling LF . Stigma: a health barrier for women with HIV/AIDS. J Assoc Nurses AIDS care. 2004;15(5):30–9. 10.1177/1055329003261981 15358923

[57] Loutfy MR , Logie CH , Zhang Y , Blitz SL , Margolese SL , Tharao WE , et al. Gender and ethnicity differences in HIV‐related stigma experienced by people living with HIV in Ontario, Canada. Wainberg M, editor. PLoS ONE. 2012;7(12):e48168. 23300514 10.1371/journal.pone.0048168 PMC3531426

[58] Simbayi LC , Kalichman S , Strebel A , Cloete A , Henda N , Mqeketo A . Internalized stigma, discrimination, and depression among men and women living with HIV/AIDS in Cape Town, South Africa. Soc Sci Med. 2007;64(9):1823–31. 17337318 10.1016/j.socscimed.2007.01.006 PMC4271649

[59] Tsai AC . Socioeconomic gradients in internalized stigma among 4,314 persons with HIV in sub‐Saharan Africa. AIDS Behav. 2015;19(2):270–82. 25572833 10.1007/s10461-014-0993-7 PMC4344381

[60] Logie C , Gadalla TM . Meta‐analysis of health and demographic correlates of stigma towards people living with HIV. AIDS Care. 2009;21(6):742–53. 19806490 10.1080/09540120802511877

[61] Nyblade L , MacQuarrie K , Phillip F , Kwesigabo G , Mbwambo J , Ndega J , et al. Working report‐ measuring HIV stigma: results of a field test in Tanzania. Washington DC: United States Agency for International Development (USAID); 2005.

[62] Mills EJ , Beyrer C , Birungi J , Dybul MR . Engaging men in prevention and care for HIV/AIDS in Africa. PLoS Med. 2012;9(2):e1001167. 22346735 10.1371/journal.pmed.1001167 PMC3274499

[63] Muula AS , Ngulube TJ , Siziya S , Makupe CM , Umar E , Prozesky HW , et al. Gender distribution of adult patients on highly active antiretroviral therapy (HAART) in Southern Africa: a systematic review. BMC Public Health. 2007;7(1):63. 17459154 10.1186/1471-2458-7-63 PMC1868718

[64] Parker R , Aggleton P . HIV and AIDS‐related stigma and discrimination: a conceptual framework and implications for action. Soc Sci Med. 2003;57(1), 13–24. 12753813 10.1016/s0277-9536(02)00304-0

[65] MacQueen KM . Framing the social in biomedical HIV prevention trials: a 20‐year retrospective. J Int AIDS Soc. 2011;14 Suppl 2:S3. 10.1186/1758-2652-14-S2-S3 PMC3194162 21968079

[66] Hayes RJ , Donnell D , Floyd S , Mandla N , Bwalya J , Sabapathy K , et al. Effect of universal testing and treatment on HIV incidence — HPTN 071 (PopART). N Engl J Med. 2019;381(3):207–18. 31314965 10.1056/NEJMoa1814556 PMC6587177

[67] Simwinga M , Bond V , Makola N , Hoddinott G , Belemu S , White R , et al. Implementing community engagement for combination prevention: Lessons learnt from the first year of the HPTN 071 (PopART) Community‐Randomized study. Current HIV/AIDS Reports. 2016;13(4):194–201. 27405816 10.1007/s11904-016-0322-z

[68] Hasan MT , Nath SR , Khan NS , Akram O , Gomes TM , Rashid SF . Internalized HIV/AIDS‐related stigma in a sample of HIV‐positive people in Bangladesh. J Heal Popul Nutr. 2012;30(1):22–30. 10.3329/jhpn.v30i1.11272 PMC3312356 22524116

[69] Logie C , Ll James , Tharao W , Loutfy M . Associations between HIV‐related Stigma, racial discrimination, gender discrimination, and depression among HIV‐positive African, Caribbean, and black women in Ontario, Canada. AIDS Patient Care STDS. 2013;27(2):114–22. 23373665 10.1089/apc.2012.0296

[70] Hargreaves JR , Krishnaratne S , Mathema H , Lilleston PS , Sievwright K , Mandla N , et al. Stangl A on behalf of the H 071 (PopART) ST. Individual and community‐level risk factors for HIV stigma in 21 Zambian and South African communities: analysis of data from the HPTN 071 (PopART) study. AIDS. 2018;32(6):783. 29369164 10.1097/QAD.0000000000001757 PMC5854764

[71] Nyblade L , Stockton MA , Giger K , Bond V , Ekstrand ML , Lean RM , et al. Stigma in health facilities: why it matters and how we can change it. BMC Med. 2019;17(1):25. 30764806 10.1186/s12916-019-1256-2 PMC6376713

[72] Wouters E , Rau A , Engelbrecht M , Uebel K , Siegel J , Masquillier C , et al. The development and piloting of parallel scales measuring external and internal hiv and tuberculosis stigma among healthcare workers in the free state province. South Africa. Clin Infect Dis. 2016;62 Suppl 3:S244–54. 27118854 10.1093/cid/civ1185 PMC5262473

[73] Sharma M , Barnabas RV , Celum C . Community‐based strategies to strengthen men's engagement in the HIV care cascade in sub‐Saharan Africa. PLoS Med. 2017;14(4):e1002262. 28399122 10.1371/journal.pmed.1002262 PMC5388461

