# Supplementary material for: Development of parallel measures to assess HIV stigma and discrimination among people living with HIV, community members and health workers in the HPTN 071 (PopART) trial in Zambia and South Africa
Source: J Int AIDS Soc. 2019 Dec 16;22(12):e25421. doi: 10.1002/jia2.25421 (PMC6912047; doi:10.1002/jia2.25421)

Supplemental Figure 1. Flowcharts for community members, health workers and people living with HIV.


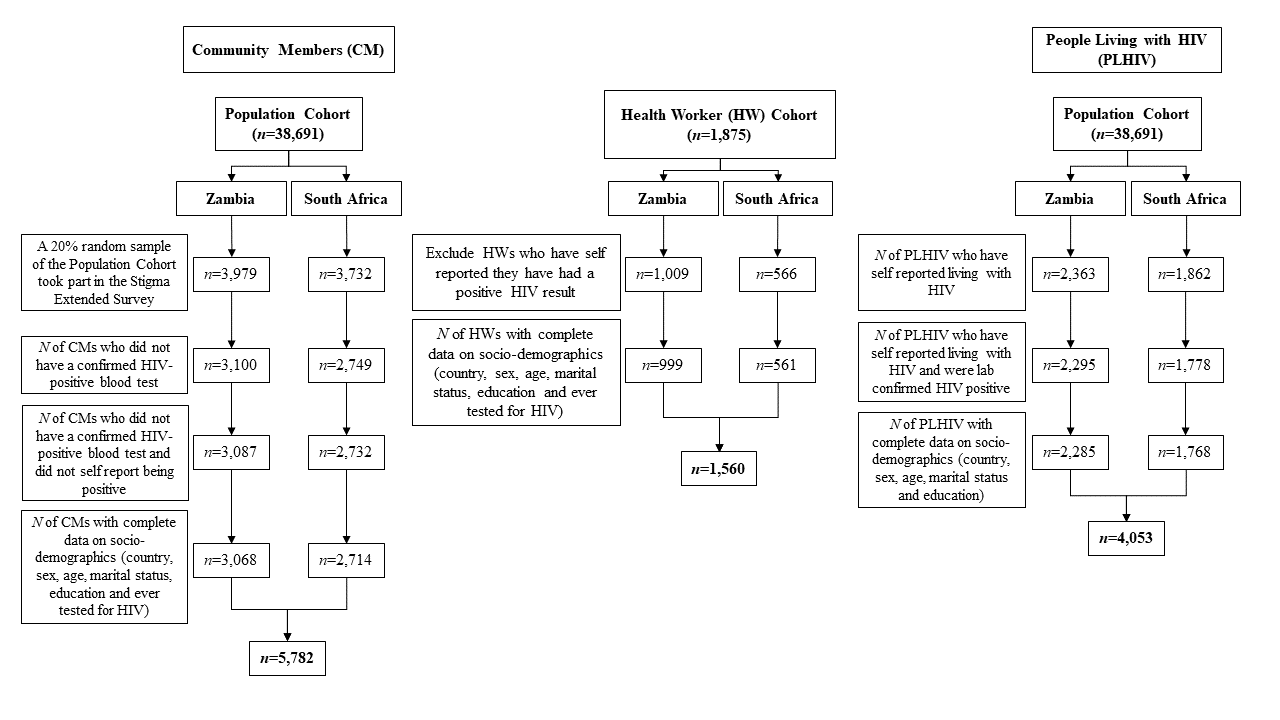

Supplement: Supplementary file 1 — Figure S1. Flowcharts for community members, health workers and people living with HIV. [file JIA2-22-e25421-s001.docx]
