# Supplementary material for: Development of parallel measures to assess HIV stigma and discrimination among people living with HIV, community members and health workers in the HPTN 071 (PopART) trial in Zambia and South Africa
Source: J Int AIDS Soc. 2019 Dec 16;22(12):e25421. doi: 10.1002/jia2.25421 (PMC6912047; doi:10.1002/jia2.25421)
Supplement: Supplementary file 2 — Table S1. Factor loadings from factor analyses for community members participating in the PopART (HPTN 071) trial in South Africa and Zambia, by country and sex Table S2. Factor loadings from factor analyses for health workers participating in the PopART (HPTN 071) trial in South Africa and Zambia, by country, sex and type of health worker Table S3. Factor loadings from factor analyses for PLHIV participating in the PopART (HPTN 071) trial in South Africa and Zambia, by country and sex. [file JIA2-22-e25421-s002.docx]

**Supplemental Table 1. Factor loadings from factor analyses for community members participating in the PopART (HPTN 071) trial in South Africa and Zambia, by country and gender.**

|  | **Zambia** | | **South Africa** | | **Men** | | **Women** | |
| --- | --- | --- | --- | --- | --- | --- | --- | --- |
| **Scale items (*stigma domain*)** | **Final Factor Loadings** | **Mean score (SD)** | **Final Factor Loadings** | **Mean score (SD)** | **Final Factor Loadings** | **Mean score (SD)** | **Final Factor Loadings** | **Mean score (SD)** |
| *Fear and judgement* |  | 0.77 (0.58) |  | 0.86 (0.58) |  | 0.83 (0.59) |  | 0.80 (0.58) |
| I fear that I could contract HIV if I come into contact with the saliva of a person living with HIV (fear) | 0.553 | 0.87 (0.79) | 0.819 | 0.90 (0.68) | 0.690 | 0.91 (0.75) | 0.652 | 0.88 (0.74) |
| I would not like to sit close to someone living with HIV, for example on public transport, at church or in a waiting room (fear) | 0.786 | 0.69 (0.74) | 0.915 | 0.84 (0.67) | 0.837 | 0.78 (0.73) | 0.846 | 0.75 (0.70) |
| I would be ashamed if someone in my family had HIV (shame) | 0.519 | 0.75 (0.76) | 0.606 | 0.83 (0.68) | 0.547 | 0.81 (0.74) | 0.565 | 0.77 (0.72) |
| Cronbach’s alpha (α) and sample size | **(α = 0.643, n = 2,883)** | | **(α = 0.818, n = 2,532)** | | **(α = 0.727, n = 1,806)** | | **(α = 0.723, n = 3,609)** | |
| *Perceived stigma in the community* |  | 1.26 (0.64) |  | 1.21 (0.69) |  | 1.26 (0.66) |  | 1.23 (0.66) |
| People sometimes talk badly about people thought to be living with HIV to others | 0.597 | 1.68 (0.93) | 0.778 | 1.38 (0.90) | 0.674 | 1.54 (0.92) | 0.671 | 1.54 (0.93) |
| People thought to be living with HIV lose respect and standing | 0.742 | 1.19 (0.86) | 0.867 | 1.17 (0.81) | 0.805 | 1.21 (0.85) | 0.801 | 1.17 (0.83) |
| People thought to be living with HIV are verbally insulted, harassed and/or threatened | 0.773 | 1.07 (0.81) | 0.905 | 1.15 (0.79) | 0.821 | 1.15 (0.81) | 0.840 | 1.09 (0.80) |
| People thought to be living with HIV are sometimes physically assaulted | 0.600 | 0.88 (0.75) | 0.771 | 1.05 (0.73) | 0.635 | 0.96 (0.73) | 0.687 | 0.96 (0.76) |
| People sometimes disclose that other people are HIV positive without their permission | 0.592 | 1.50 (0.94) | 0.682 | 1.27 (0.86) | 0.659 | 1.43 (0.89) | 0.611 | 1.38 (0.92) |
| Cronbach’s alpha (α) and sample size | **(α = 0.791, n = 2,803)** | | **(α = 0.897, n = 2,453)** | | **(α = 0.840, n = 1,757)** | | **(α = 0.841, n = 3,499)** | |
| *Perceived stigma in the health setting* |  | 0.92 (0.72) |  | 1.13 (0.74) |  | 1.07 (0.75) |  | 0.99 (0.73) |
| Health workers sometimes talk badly about people living with or thought to be living with HIV to others | 0.769 | 0.93 (0.82) | 0.799 | 1.15 (0.82) | 0.799 | 1.10 (0.85) | 0.780 | 1.00 (0.82) |
| Health workers sometimes disclose that other people are HIV positive without their permission | 0.769 | 0.91 (0.80) | 0.799 | 1.11 (0.81) | 0.799 | 1.03 (0.81) | 0.780 | 0.99 (0.81) |
| Cronbach’s alpha (α) and sample size | **(α = 0.743, n = 2,842)** | | **(α = 0.779, n = 2,464)** | | **(α = 0.779, n = 1,767)** | | **(α = 0.757, n = 3,539)** | |
| *Items dropped from final scale* |  |  |  |  |  |  |  |  |
| People are hesitant to take an HIV test due to fear of other people’s reaction if the test result is positive (anticipated stigma) ^a^ |  | 1.83 (1.00) |  | 1.47 (0.94) |  | 1.68 (0.98) |  | 1.65 (0.99) |
| PLHIV who are taking ART are treated better by others than PLHIV who are not taking ART (anticipated stigma) ^b^ |  | 1.16 (0.90) |  | 0.94 (0.68) |  | 1.09 (0.79) |  | 1.04 (0.82) |

SD: Standard deviation

^a^: n=2,935 in Zambia, n=2,582 in South Africa, n=1,847 in men, n=3,670 in women.

^b^: n=2,734 in Zambia, n=2,419 in South Africa, n=1,721 in men, n=3,432 in women.

**Supplemental Table 2. Factor loadings from factor analyses for health workers participating in the PopART (HPTN 071) trial in South Africa and Zambia, by country, gender and type of health worker.**

|  | **Zambia** | | **South Africa** | | **Men** | | **Women** | | **HFS** | | **CHiPs** | | **CCW** | |
| --- | --- | --- | --- | --- | --- | --- | --- | --- | --- | --- | --- | --- | --- | --- |
| **Scale items (*stigma domain*)** | **Final Factor Loadings** | **Mean**  **score (SD)** | **Final Factor Loadings** | **Mean**  **score (SD)** | **Final Factor Loadings** | **Mean**  **score (SD)** | **Final Factor Loadings** | **Mean**  **score (SD)** | **Final Factor Loadings** | **Mean**  **score (SD)** | **Final Factor Loadings** | **Mean**  **score (SD)** | **Final Factor Loadings** | **Mean score (SD)** |
| *Fear and judgement* |  | 0.78 (0.49) |  | 0.78 (0.49) |  | 0.80 (0.50) |  | 0.77 (0.49) |  | 0.76 (0.52) |  | 0.74 (0.42) |  | 0.97 (0.50) |
| HIV is punishment from God | 0.499 | 0.60 (0.74) | 0.468 | 0.54 (0.69) | 0.408 | 0.59 (0.77) | 0.520 | 0.58 (0.70) | 0.536 | 0.57 (0.73) | 0.462 | 0.53 (0.68) | 0.309 | 0.75 (0.74) |
| I would be ashamed if someone in my family had HIV | 0.668 | 0.57 (0.66) | 0.728 | 0.59 (0.67) | 0.670 | 0.60 (0.66) | 0.694 | 0.57 (0.66) | 0.678 | 0.58 (0.69) | 0.746 | 0.50 (0.58) | 0.657 | 0.74 (0.73) |
| I avoid physical contact with clients living with HIV | 0.480 | 0.81 (0.84) | 0.494 | 0.85 (0.82) | 0.512 | 0.82 (0.84) | 0.480 | 0.82 (0.83) | 0.519 | 0.77 (0.83) | 0.362 | 0.79 (0.80) | 0.484 | 1.09 (0.86) |
| Other people deserve access to health services more than PLHIV | 0.504 | 0.87 (0.85) | 0.454 | 0.95 (0.81) | 0.502 | 0.93 (0.86) | 0.473 | 0.89 (0.83) | 0.640 | 0.77 (0.79) | 0.259 | 1.02 (0.88) | 0.452 | 1.10 (0.86) |
| I fear that I could contract HIV if I come into contact with the saliva of a person living with HIV | 0.314 | 1.07 (0.83) | 0.497 | 0.96 (0.81) | 0.435 | 1.07 (0.85) | 0.349 | 1.01 (0.82) | 0.364 | 1.09 (0.88) | 0.283 | 0.87 (0.69) | 0.493 | 1.20 (0.85) |
| Cronbach’s alpha (α) and sample size | **(α = 0.604, n = 909)** | | **(α = 0.650, n = 453)** | | **(α = 0.620, n = 382)** | | **(α = 0.617, n = 980)** | | **(α = 0.671, n = 734)** | | **(α = 0.482, n = 444)** | | **(α = 0.592, n = 184)** | |
| *Perceived co-worker stigma* |  | 1.00 (0.60) |  | 0.78 (0.59) |  | 1.01 (0.62) |  | 0.89 (0.60) |  | 0.94 (0.60) |  | 0.87 (0.62) |  | 0.97 (0.60) |
| My co-workers sometimes gossip about clients’ HIV test results | 0.631 | 1.21 (0.85) | 0.722 | 0.84 (0.79) | 0.622 | 1.21 (0.86) | 0.689 | 1.04 (0.84) | 0.655 | 1.10 (0.86) | 0.702 | 1.09 (0.87) | 0.714 | 1.00 (0.76) |
| My co-workers sometimes talk badly about people thought to be living with HIV | 0.556 | 1.25 (0.85) | 0.638 | 0.99 (0.80) | 0.572 | 1.20 (0.84) | 0.605 | 1.15 (0.84) | 0.578 | 1.21 (0.83) | 0.619 | 1.06 (0.84) | 0.598 | 1.21 (0.87) |
| My co-workers sometimes treat PLHIV poorly when providing them with health services | 0.728 | 0.85 (0.76) | 0.822 | 0.66 (0.68) | 0.761 | 0.88 (0.79) | 0.760 | 0.74 (0.71) | 0.755 | 0.80 (0.74) | 0.786 | 0.72 (0.73) | 0.713 | 0.84 (0.72) |
| My co-workers sometimes verbally insult clients living with HIV | 0.665 | 0.69 (0.74) | 0.757 | 0.62 (0.66) | 0.687 | 0.73 (0.78) | 0.684 | 0.64 (0.69) | 0.676 | 0.66 (0.70) | 0.674 | 0.61 (0.71) | 0.744 | 0.81 (0.75) |
| Cronbach’s alpha (α) and sample size | **(α = 0.736, n = 946)** | | **(α = 0.819, n = 499)** | | **(α = 0.753, n = 401)** | | **(α = 0.774, n = 1,044)** | | **(α = 0.757, n = 779)** | | **(α = 0.758, n = 460)** | | **(α = 0.781, n = 206)** | |
| *Perceived stigma in the community* |  | 1.45 (0.51) |  | 1.60 (0.48) |  | 1.52 (0.50) |  | 1.49 (0.51) |  | 1.41 (0.52) |  | 1.59 (0.45) |  | 1.64 (0.49) |
| People thought to be living with HIV are sometimes physically assaulted | 0.452 | 1.08 (0.78) | 0.446 | 1.22 (0.76) | 0.394 | 1.13 (0.78) | 0.479 | 1.13 (0.78) | 0.460 | 1.07 (0.77) | 0.403 | 1.14 (0.76) | 0.545 | 1.34 (0.81) |
| People hesitate to start ARV drugs because they are afraid others will learn they are living with HIV | 0.439 | 1.92 (0.83) | 0.459 | 2.02 (0.80) | 0.526 | 1.94 (0.78) | 0.423 | 1.96 (0.84) | 0.478 | 1.87 (0.86) | 0.432 | 2.08 (0.74) | 0.282 | 1.98 (0.81) |
| People sometimes talk badly about people thought to be living with HIV | 0.525 | 1.93 (0.77) | 0.421 | 2.06 (0.69) | 0.568 | 1.97 (0.72) | 0.472 | 1.98 (0.75) | 0.524 | 1.86 (0.79) | 0.433 | 2.12 (0.67) | 0.363 | 2.10 (0.68) |
| People thought to be living with HIV are verbally insulted, harassed or threatened | 0.614 | 1.15 (0.78) | 0.692 | 1.38 (0.81) | 0.644 | 1.28 (0.80) | 0.644 | 1.21 (0.79) | 0.662 | 1.09 (0.76) | 0.606 | 1.37 (0.81) | 0.586 | 1.43 (0.81) |
| People thought to be living with HIV lose respect and standing | 0.573 | 1.16 (0.81) | 0.477 | 1.31 (0.76) | 0.494 | 1.28 (0.81) | 0.572 | 1.19 (0.79) | 0.603 | 1.15 (0.79) | 0.401 | 1.25 (0.77) | 0.600 | 1.35 (0.83) |
| Cronbach’s alpha (α) and sample size | **(α = 0.648, n = 940)** | | **(α = 0.623, n = 465)** | | **(α = 0.652, n = 395)** | | **(α = 0.644, n = 1,010)** | | **(α = 0.675, n = 755)** | | **(α = 0.564, n = 448)** | | **(α = 0.592, n = 202)** | |
| *Items dropped from final scale* |  |  |  |  |  |  |  |  |  |  |  |  |  |  |
| Health workers living with HIV do not disclose their status at work for fear of losing their job^a^ |  | 1.13 (0.92) |  | 1.39 (0.97) |  | 1.19 (0.91) |  | 1.22 (0.96) |  | 1.18 (0.94) |  | 1.22 (0.95) |  | 1.35 (0.97) |
| People get HIV because they engage in irresponsible behaviors^b^ |  | 1.13 (0.87) |  | 1.10 (0.87) |  | 1.32 (0.91) |  | 1.04 (0.84) |  | 1.13 (0.86) |  | 1.02 (0.85) |  | 1.30 (0.92) |
| If someone asks for an HIV test, they have probably behaved irresponsibly^c^ |  | 1.25 (0.89) |  | 1.28 (0.88) |  | 1.27 (0.88) |  | 1.25 (0.89) |  | 1.18 (0.90) |  | 1.27 (0.87) |  | 1.51 (0.83) |

CCW: Community-based lay health workers; CHiP: Community HIV-care Providers; HFS: Health facility staff; SD: Standard deviation

^a^: n=967 in Zambia, n=506 in South Africa, n=404 in men, n=1069 in women, n=791 in health facility staff, n=473 in CHiPs, n=209 in CCW.

^b^: n=982 in Zambia, n=529 in South Africa, n=416 in men, n=1095 in women, n=808 in health facility staff, n=488 in CHiPs, n=215 in CCW.

^c^: n=979 in Zambia, n=533 in South Africa, n=412 in men, n=1100 in women, n=809 in health facility staff, n=493 in CHiPs, n=210 in CCW.

**Supplemental Table 3. Factor loadings from factor analyses for PLHIV participating in the PopART (HPTN 071) trial in South Africa and Zambia, by country and gender.**

|  | **Zambia** | | **South Africa** | | **Men** | | **Women** | |
| --- | --- | --- | --- | --- | --- | --- | --- | --- |
| **Scale items (*stigma domain*)** | **Final Factor Loadings** | **Mean score (SD)** | **Final Factor Loadings** | **Mean score (SD)** | **Final Factor Loadings** | **Mean score (SD)** | **Final Factor Loadings** | **Mean score (SD)** |
| *Internalized Stigma* |  | 0.80 (0.67) |  | 0.89 (0.58) |  | 0.85 (0.65) |  | 0.84 (0.63) |
| I have lost respect or standing in the community because of my HIV status | 0.639 | 0.79 (0.81) | 0.701 | 0.88 (0.63) | 0.651 | 0.83 (0.79) | 0.662 | 0.83 (0.73) |
| I think less of myself because of my HIV status | 0.870 | 0.81 (0.78) | 0.947 | 0.91 (0.67) | 0.877 | 0.88 (0.76) | 0.903 | 0.85 (0.74) |
| I have felt ashamed because of my HIV status | 0.722 | 0.80 (0.81) | 0.839 | 0.89 (0.66) | 0.758 | 0.83 (0.75) | 0.766 | 0.84 (0.75) |
| Cronbach’s alpha (α) and sample size | **(α = 0.784, n = 2,193)** | | **(α = 0.867, n = 1,740)** | | **(α = 0.802, n = 463)** | | **(α = 0.818, n = 3,470)** | |

SD: Standard deviation
